# A Database of Domain Definitions for Proteins with Complex Interdomain Geometry

**DOI:** 10.1371/journal.pone.0005084

**Published:** 2009-04-08

**Authors:** Indraneel Majumdar, Lisa N. Kinch, Nick V. Grishin

**Affiliations:** 1 Howard Hughes Medical Institute, University of Texas Southwestern Medical Center at Dallas, Dallas, Texas, United States of America; 2 Department of Biochemistry, University of Texas Southwestern Medical Center at Dallas, Dallas, Texas, United States of America; Center for Genomic Regulation, Spain

## Abstract

Protein structural domains are necessary for understanding evolution and protein folding, and may vary widely from functional and sequence based domains. Although, various structural domain databases exist, defining domains for some proteins is non-trivial, and definitions of their domain boundaries are not available. Here, we present a novel database of manually defined structural domains for a representative set of proteins from the SCOP “multi-domain proteins” class. (http://prodata.swmed.edu/multidom/). We consider our domains as mobile evolutionary units, which may rearrange during protein evolution. Additionally, they may be visualized as structurally compact and possibly independently folding units. We also found that representing domains as evolutionary and folding units do not always lead to a unique domain definition. However, unlike existing databases, we retain and refine these “alternate” domain definitions after careful inspection of structural similarity, functional sites and automated domain definition methods. We provide domain definitions, including actual residue boundaries, for proteins that well known databases like SCOP and CATH do not attempt to split. Our alternate domain definitions are suitable for sequence and structure searches by automated methods. Additionally, the database can be used for training and testing domain delineation algorithms. Since our domains represent structurally compact evolutionary units, the database may be useful for studying domain properties and evolution.

## Introduction

Although protein domains commonly represent units of protein function [1] they can also be visualized as structurally compact semi-independent building blocks [2], [3]. Hence, historically, various criteria have been used in defining domains; namely function, sequence, evolution, structure, and folding considerations. Domain definitions based on these various considerations do not always agree with each other. For instance, a structurally compact unit might not correspond to a unit of known biological function [4], as functional sites are frequently housed between structural domains. In this article we present a database of structurally defined protein domains with due consideration to evolutionary mechanisms. Our selected structures include some of the most structurally complex proteins known and are catalogued in the “multi-domain proteins” class of SCOP [5]. Reliable manual databases like SCOP and CATH [6] provide residue boundaries only for domains in some of these structures.

Domains are structurally characterized by the presence of isolated hydrophobic cores. Additionally, intra-domain residue contacts are more extensive than between domains [1], [7], [8]. This discrepancy in residue contacts can indicate nucleation regions during the folding process. Thus, structural domains may also be referred to as independent folding units [9]. Alternatively, during protein evolution, modular rearrangements of the primary sequence by insertions or deletions may occur [10], [11]. These evolutionary modules may also be visualized as domains and can be defined based on co-occurrence with other domains in different proteins [12], [13]. A logical extension of this modularity is the maintenance of sequence continuity of domains. Our work utilizes both these viewpoints in order to define domains.

One key benefit of a reliable set of reference domains is in similarity searches where the reference domains can be used as a query. Secondly, they can also be a training set for automated domain definition method development. Such reference domains are usually obtained from existing databases like SCOP [5] or CATH [6]. SCOP provides a manually curated protein classification database of domains defined largely by homology. For example, if a segment of polypeptide chain is present in several proteins, but is joined with different, non-homologous segments, it is considered a domain. Similarly CATH provides hierarchical domain classification based on architecture (overall shape), topology (folding) and homology but utilizes a combination of automated and manual procedures. A number of automated methods are also available for defining domains. The more reliable ones use similarity detection, especially by primary and tertiary structure searches using a query domain. However, using these similarities to locate related structures only works well for larger domains or for higher identity [14], [15], and for which reliable query domain-definitions are available. In this article we compare our domain definitions with those obtained from the automated methods Domak [16] and PDP [17]. Both methods use inter-residue contacts to locate compact structural regions. Additionally, Domak uses secondary structure packing and statistical parameters derived from a set of reference domains, as described in literature, for domain delineation. Alternatively, PDP decomposes domains by minimizing chain break between spatially close residues. However, in spite of the large array of existing databases and automated methods, agreement in domain definitions between them has been observed only for the simplest protein topologies [18].

As a result of this work, we developed a database of manual domain definitions with assigned residue ranges based primarily on the combined concepts of structural similarity, compactness and sequence continuity. These domain definitions are available online at http://prodata.swmed.edu/multidom/. Our domain definitions will be helpful not only for similarity searches and development of automated methods but possibly also for studying domain properties and evolution.

## Results

We provide a novel database of manually defined domains, with residue boundaries for each domain, for a set of topologically complex proteins (http://prodata.swmed.edu/multidom/). Our domain definitions are based on structural, functional, sequence and evolutionary considerations after careful study of relevant literature and inspection of domains defined by existing automated methods. We provide downloadable PyMol [19] scripts to easily view our domain definitions for each chain, as well as domain sequences and 3D coordinates for every domain. The domain definitions are for a representative set (40% sequence identity, 157 total chains) of PDB [20] chains from the “multi-domain proteins” class in SCOP (version 1.73) [5]. PDB chains in the database were classified into 53 groups of homologous proteins. These groups correspond to SCOP “folds” in the “multi-domain protein” class. Larger and more diverse groups (e.g. polymerases) were split into sub-groups.

### Alternate domain definitions

Domains in our database represent compact evolutionary modules that can fuse terminally to or insert within the primary sequence of other domains. We consider this fusion or insertion of a domain to be caused by a single evolutionary event. For some proteins, this evolutionary consideration of modular domains could support alternative domain definitions (alternate evolutionary modules). Based on structural overlap of terminal extensions, the same pair of domains could be visualized as being either terminally fused (sequence continuous) or inserted one within the other (sequence discontinuous). For proteins with a large number of domains such an alternate definition might be possible with regard to only a few, or even just a pair, of the constituent domains. Additionally, domain-modularity concepts bar certain structurally compact regions from having arisen by a single evolutionary event. Typically these consist of a structurally compact group of inserts into other domains. Rather than omitting these regions from our database, we define them as yet another alternate domain definition (composite domains). These alternate domain definitions are explained below.

#### Alternate evolutionary modules

**Figure 1:**
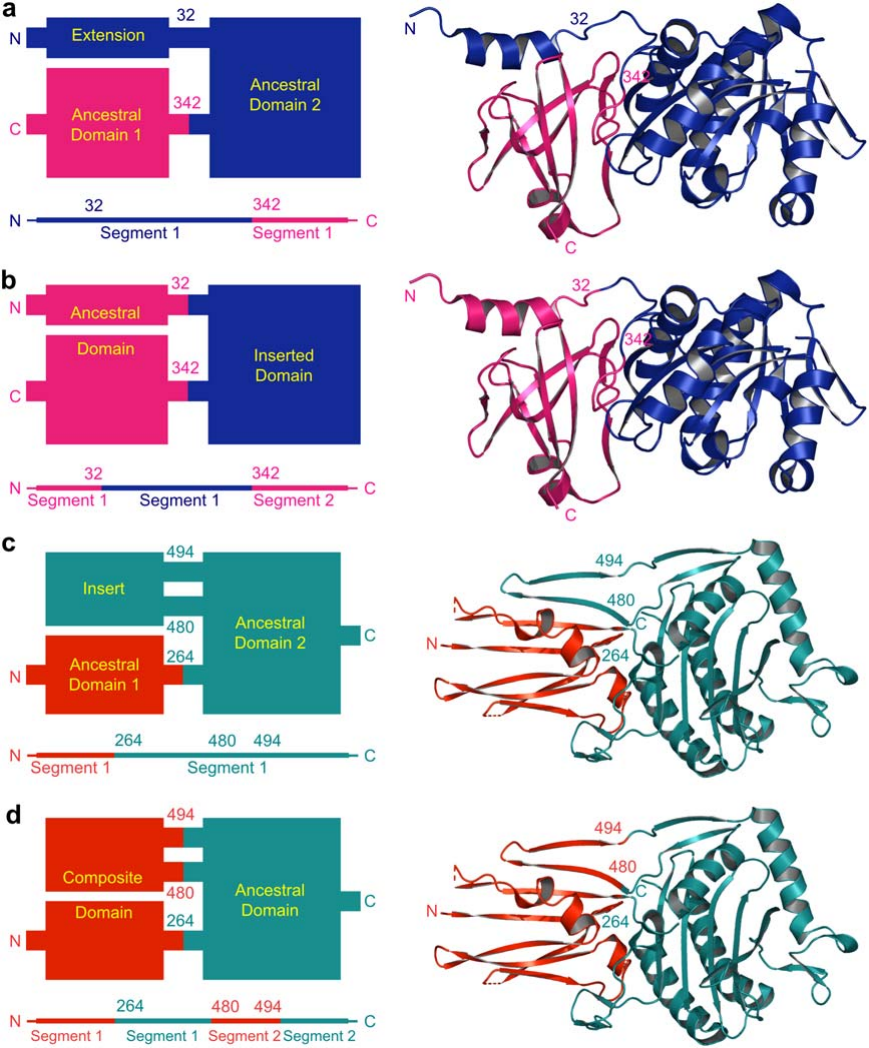
Domain Definition Categories. Block-diagram domain-architecture schematics representing the strategy for domain definition categories are shown on the left, with the corresponding structures (1amu for a and b; 1qme for c and d) on the right. A schematic sequence-view representing the position of domains in the polypeptide chain is shown below each block-diagram. Residue numbers are marked at linkers joining domains, with N and C marking the termini. Only a part of the protein structure and corresponding schematics are shown for clarity. Broken lines indicate domains omitted from the structures. Terminal extensions that protrude from one domain yet interact with another domain are defined (a) by sequence proximity (“by sequence”) or (b) by structure proximity (“by structure”). Protruding domain insertions that interact with neighboring domains are defined (c) by sequence proximity or by (d) structural proximity resulting in a composite domain.

A common occurrence in multi-domain protein structures was the presence of relatively short non-globular extensions at the N- or the C-terminus of a domain that interacted with a neighboring domain. Such terminal extensions are known to stabilize associated domains (fig 1a blue N-terminal α-helical extension from ancestral domain 2 interacts with fused pink ancestral domain 1). This scenario resulted in a continuous sequence for each domain (fig 1a blue segment followed by pink segment). Alternatively, the extensions could result from a domain insertion event near the termini of an ancestral domain (fig 1b blue inserted domain splits pink ancestral domain), producing a sequence discontinuity in the ancestral domain (fig 1b blue segment splits pink segment). Because each of these scenarios is equally possible in evolutionary terms, terminal extensions are defined separately, and alternative domain definitions are provided: “By sequence” definitions include the extension as part of the sequence continuous domain (fig 1a), while “by structure” definitions include the extension as part of the structurally interacting domain (fig 1b).

#### Composite domains

In addition to terminal extensions, some domains included a relatively short non-globular extension that protruded from within the domain (a non-terminal extension) yet interacted with another domain. For example, a β-hairpin extending from the middle of a profillin-like α/β/α sandwich domain in penicillin-binding protein 2× extends the β-sheet of an N-terminal domain (fig 1c and d). This β-hairpin probably arose as an insertion to the profillin domain that stabilizes pre-existing interactions with the N-terminal domain (fig 1c teal insert to ancestral domain 2 interacts with red ancestral domain 1). Retaining the insertion with the protruding domain keeps sequence continuity for both domains (fig 1c red segment followed by teal segment) and obeys evolutionary assumptions of domain modularity. Alternatively, the insertion could be defined structurally as belonging to the N-terminal domain with which it interacts. Such an assignment resulted in a “composite” domain that could only be explained by multiple insertion events and resulted in sequence discontinuity (fig 1d red insertion into teal domain completes red composite domain). Additionally, some neighboring non-globular domains were also observed to form compact globular units with shared hydrophobic cores. Our database also provides composite domain definitions for these domain clusters. Thus, the multi-domain protein dataset included composite domains consisting of various combinations of insertions and domains (insertion+insertion, insertion+domain, or domain+domain).

In summary, our database provides four categories of definitions: “By structure”, “by sequence” and composite domains, and extensions. The “by structure” definitions are more applicable to structure similarity searches, as the terminal extensions associated with the spatially closest domain may be important in finding remote homologs. “By-sequence” definitions that attribute terminal extensions to the sequence-continuous polypeptide segment (similar to SCOP) are more useful for sequence search strategies; as such regions may contain conserved sequence motifs. The “extensions” category specifies extended regions (terminal extensions, insertions and linkers) that differentiate between the “by structure” and “by sequence” definitions. Finally, “composite” domains do not represent evolutionary units and are purely geometric, but may be useful in studies of convergent evolution.

### Comparison with other manual and automated domain definition methods

**Figure 2:**
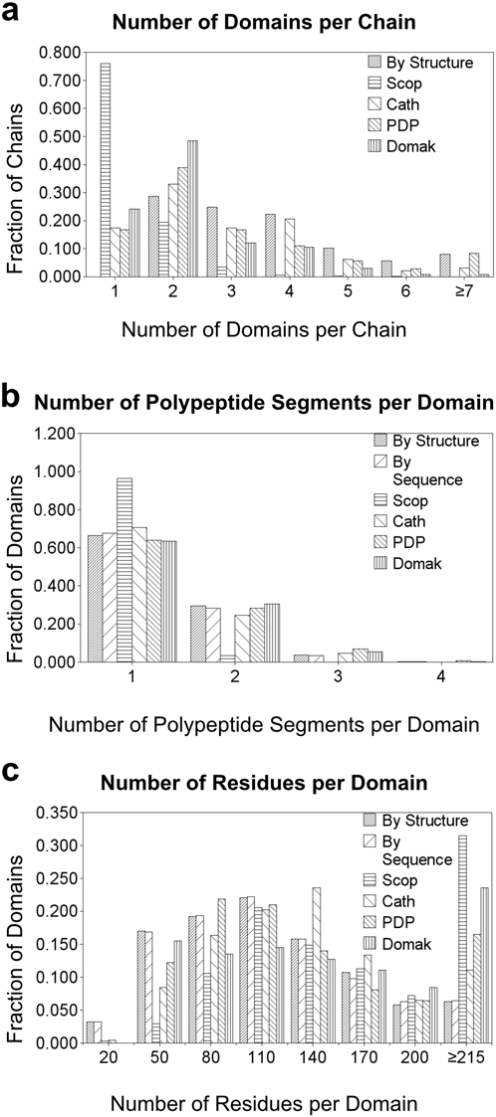
Domain Definition Comparisons. “By Structure” and “By Sequence” category definitions (see fig 1a, b) are compared with CATH and automated methods “PDP” and “DOMAK” for our *structure dataset* (see methods). Data for SCOP is generated from PDB chains in SCOP classes 1 through 4. Data on the vertical axis is normalized to the total number of PDB chains or domains in the respective dataset. (a) Number of domains defined per chain by each method. Data for “By Sequence” is identical to “By Structure” and is not shown. (b) Number of polypeptide segments comprising each domain. (c) Histogram representing residue length of defined domains. Only domains up to 215 residues long are shown for clarity.

Manually defined domains in our database are of three alternate categories (613 “by structure”, 612 “by sequence”, and 58 “composite”). Additionally, the database separately defines 83 inserts, linkers and terminal extensions that also are part of the domain definitions. CATH [6], which is a database created using manual and automated procedures, and two automated methods, namely PDP [17] and Domak [16], defined 273, 443 and 297 domains, respectively, for the structures in our database. However, domains for 58 PDB chains (out of 157) were not yet assigned by CATH when this project was completed. We defined at least two domains for every chain in the multi-domain proteins class (fig 2a by structure), with the number of domains defined per chain falling gradually from 2 to 6. Manual definitions also split 13 chains into 7 or more domains, and are mostly proteins involved in replication and transcription, notably polymerases. Although SCOP [5] did not always provide domain ranges for multi-domain class comparison, the database rarely defines more than three domains per chain in the first four structure classes (fig 2a SCOP). CATH domain numbers were closest to our manual method (fig 2a CATH), with the number of chains having 2–6 domains being very similar. However, CATH did define 14 chains as single domains, and few chains with 7 or more domains. Automated domain definition methods, especially DOMAK (fig 2a DOMAK), tended to assign fewer domains. PDP performed close to manual definitions, however, with less number of domains per chain (fig 2a PDP). Unlike our definitions, PDP did define chains as single domains, whereas number of chains with 7 or more domains was very similar.

Despite more domains being defined manually for a single chain in contrast to automated methods, manual definition produced a marginally lower number of polypeptide segments per domain (fig 2b). This increase in sequence continuity of manual domains results from imposing evolutionary assumptions on the splits: Compact regions composed of multiple inserted segments could not represent single evolutionary events and were treated in a special category of “composite” domains. The difference in the number of polypeptide segments present per domain between the manual “by sequence” and “by structure” category definitions indicated structures that contained terminal extensions. Hence, domains where terminal extensions were assigned sequence continuous (by sequence) have a slightly lower number of segments than domains where terminal extensions were assigned by structural proximity (by structure). In contrast to both manual and automated domain definitions, SCOP rarely introduced sequence discontinuity in its classification of the first four structure classes (fig 2b SCOP). CATH defined slightly higher number of segments per domain than our definitions.

For analysis of domain size, our manual definitions were comparable to PDP, SCOP and CATH in the percentage of defined domains ranging between 95 and 215 residues in length, with DOMAK shifting towards longer domains (fig 2c). However, within this range, CATH defined more domains with length ranging from 125–185 residues. Whereas all methods showed a peak at around 95 residues per domain, CATH showed a peak at around 140 followed by a sudden drop in domain size. In contrast to others, our database included a significant number of domains with less than 50 residues and rarely defined domains longer than 300 residues. Our manual approach identified very short domains (e.g. zinc-fingers found by inspection of cysteines and histidines) that automated methods did not detect. Additionally, our definitions tended towards providing smaller compact domains.

During our visual assessment, we found that automated methods failed to provide consistent domain definitions for most chains in the dataset. Automated methods reached a consensus only on very simple cases, where structurally compact domains displayed few inter domain interactions. In almost every case, the domain boundaries from these programs required further refinement, even if the domain number and their general locations were correct. Domain definitions obtained from CATH, although incomplete, were more reliable. However we did notice stray inconsistencies in domain definitions between similar proteins. These problems and limitations of domain definition were well documented by others [18]. However, we found even these limited definitions to be useful, for instance in suggesting potential domain cores. Literature helped in our functional considerations for several domain definitions, e.g. in 1ecr [21] non compact functional regions and residues at domain interfaces were analyzed, so that they could be assigned to the correct domain.

### Evolutionary considerations: An example of domain delineation in DNA/RNA polymerases

**Figure 3:**
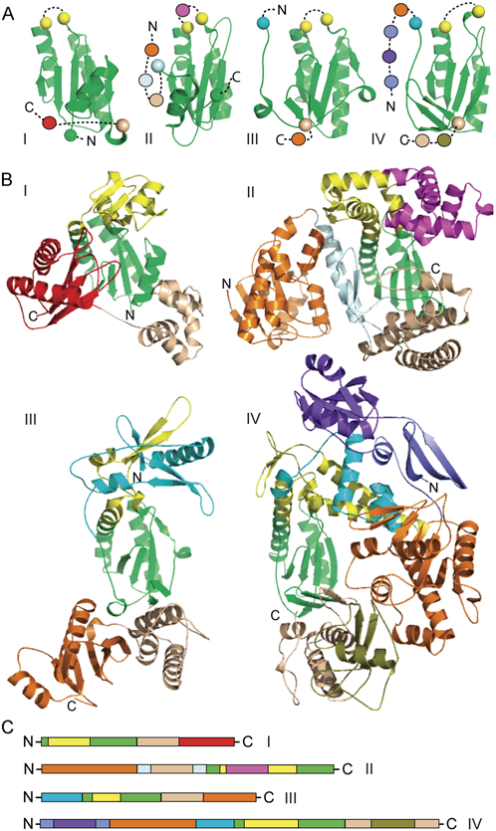
Modular Domains in Polymerases. Diverse polymerase structures displaying domain organizations of varying complexity and connectivity are divided into four labeled subgroups: i) Y family DNA polymerase, ii) Klenow DNA polymerase / T7 phage polymerases, iii) Reverse transcriptase / RNA-dependent RNA polymerase, and iv) DNA polymerase I. a) All polymerase structures possess a homologous catalytic Palm domain (green cartoon models). Palm domains from representatives of each polymerase subset are depicted from left to right in similar orientations (i 1jx4; ii 1u4b, iii 1vrt, and iv 1tgo). Colored spheres mark palm domain boundaries: inserted finger domain (yellow), N-terminal domains (cyan and light blue), or C-terminal domain (wheat). Additional domains are represented as colored spheres connected from N- to C- terminus by a dashed line. b) Cartoon structure models of *Sulfolobus solfataricus* DNA polymerase IV (i) *Bacillus stearothermophilus* DNA polymerase I, (ii), *HIV-I* reverse transcriptase (iii), and *Thermococcus gorgonarius* type B DNA polymerase (iv). Colored as in A. c) Sequence continuity of defined domains represented as blocks from N- to C-terminus.

For protein families with a large number of available structures, combining structural similarity with evolutionary considerations was especially useful while defining domains. Our modular domains could be positively identified and boundaries refined by structural similarity of conserved domains, insertion position and general topology of various associated domains, and interactions between them. We illustrate our general line of thought and difficulties with domain definition using DNA/RNA polymerases as an example. The common architecture of polymerases includes three functional domains commonly referred to as “palm”, “thumb” and “finger”, with the ubiquitous palm domain providing the catalytic activity for the enzymes [22]. We sub-divided the polymerases into four groups based on ease of structural alignment and similarity of the three common domains (fig 3, I–IV).

In polymerase structures from all groups, the catalytic palm domain incorporated a ferredoxin-like fold (fig 3a, green) that was variably decorated by a number of additional domains. The ferredoxin-like fold itself contained various additional secondary structural elements packing against the conserved β-sheet (fig 3a): The palm domain in group I included a C-terminal α/β extension, group II included an N-terminal helical extension and group III and IV included a C terminal β-hairpin. Our domains show considerable variation in size and globularity (fig 3b: cyan and orange domains). Additionally, these different domains could combine variously within a single structure. Numerous domain insertions were observed; hence, although all the domains were compact and modular, not all were sequence continuous (fig 3c). For instance, sequence discontinuity in the green palm domain was caused by insertion of the yellow fingers domain. Our evolutionary considerations could explain each of these domain insertions as arising from distinct single evolutionary events. Furthermore, “nested’ insertions were also observed and could have occurred due to a cascade of domain insertions (eg. the structurally compact magenta domain inserted into the yellow domain, where the yellow domain itself was inserted into the green domain in fig 3II).

While the palm domain was the most conserved, and hence represented an evolutionary core unit of the polymerase structures, the thumb and finger domains exhibited significant topological variation. Structural position of the thumb domain was conserved with respect to the palm (fig 3b, wheat), although its sequential placement varied (fig 3c, wheat). Thumb domain definitions were therefore based on position with respect to the palm. Group 1, III and IV thumbs were fused to the palm C-terminus, while group II thumbs were found N-terminal to the palm as a nested insertion (fig 3, light blue). Additionally, despite retaining a mainly α-helical secondary structure composition, thumb domains displayed low structural similarity between groups and included differing sizes and topologies of α-helices. In contrast to thumb domains, fingers were defined based on sequential position with respect to the palm domain. A component of the finger domain (fig 3, yellow) was always inserted into the palm domain at the same position. However, the finger domain's secondary structure composition, topology, and interactions with neighboring domains varied.

Composite domains were also observed among polymerases. Together with an N-terminal domain (figure 3, cyan), insertions from Group III and IV fingers formed a composite globular structure with extensive inter-domain interactions and a shared hydrophobic core. The composite domain was sequence discontinuous (figure 3C, cyan, green and yellow) and its formation could not be explained evolutionarily by a single insertion event. Hence, we do not consider it as an evolutionary module. Thus, composite domains represented a special case, where primary sequence arrangement and evolutionary considerations indicated two separate domains while structure suggested a combined domain definition.

Apart from the palm, thumb and fingers, polymerase structures included various additional domains. One such domain resembled a Ribonuclease H-like (RNAseH-like) fold topology (fig 3b, orange). This RNAseH-like domain was found to be variously located in the primary sequence (fig 3c, orange) among the polymerases. Structures in group II and IV included RNAseH-like domains N-terminal to the common polymerase components, while in group III the domain was C-terminal. Structures in group I all together lack an RNAseH-like domain. These variations illustrate modular rearrangement of domains among polymerases [11] and provide further clues to domain boundaries.

## Discussion

Although biologists agree that proteins are composed of domains and that analysis of constituent domains are important for studying the whole protein, there is widespread disagreement regarding the properties and definition of the individual domains themselves. Thus, the concept of a domain (what is a domain?) and the methods to delineate them in a given protein (how to find domain boundaries?) must be addressed, and are a pre-requisite for automated analysis of various protein properties. Additionally, questions arise concerning the possibility of providing consistent domain definitions for proteins based on a few general principles. This work, based on a set of topologically complex proteins, represents a step towards these important goals.

### What is a domain?

Perhaps the greatest difficulty in domain studies is the absence of a uniform answer to the question: What is a domain? Interestingly, several domain “concepts” can be proposed. Among them, the following five widespread concepts are worth noting. 1) *Functional domains* are characterized by functional independence, and form units sufficient for a certain, mainly enzymatic, activity. Since functional characterization of a protein domain is often a primary objective, protein domains are usually thought of as functional domains. 2) *Sequence domains* are regions of polypeptide chain that can be detected by sequence similarity and can be found in combination with other sequence domains. These domains are widely used in sequence analysis where incorrect boundaries imply erroneous delineation of conserved regions. Since conserved regions are relied upon by sequence-profile based search methods such as PSI-BLAST [23] and other transitive strategies for homology inference, correct delineation of domain boundaries are essential. 3) *Evolutionary domains* are modules that can shuffle between proteins via recombination, transposition, exon shuffling and other mutational events. Thus their occurrence in dissimilar domain contexts, provided homology can be detected by sequence or structural similarity, forms the basis of evolutionary domain definition. 4) *Domains as folding units* were defined in the early days of protein studies [2]. This concept implies that an isolated domain is capable of independent folding, or at least possesses a folding nucleus that can initiate protein folding. 5) *Structural domains* are defined geometrically by structural compactness, presence of a hydrophobic core and more extensive amino acid interactions intra-domain rather than inter-domain. Such domains are essential for structural similarity search. For many proteins, e.g. those that look like “beads on a string” with domains that are well separated from each other, all 5 concepts may lead to the same domain definition. However, the criteria used to formulate these five domain concepts are quite different, and bringing these concepts together consistently may not always be possible. When domains interact more closely, different concepts inevitably lead to differing definitions. This work attempts to reconcile several of these domain concepts on some of the most topologically complex examples of protein chains.

### Alternate domain definitions

Domains may be considered as evolutionary units that can potentially shuffle between proteins, and are a natural viewpoint for biodiversity studies. This evolutionary consideration provides an easy guide to domain identification when sequence similarity between domains from different proteins is high, even if the domain structures differ. However, when sequence or even structural similarity to other proteins is harder to ascertain, domain definition becomes especially difficult. This difficulty is particularly noticeable at domain boundaries, where domain definition tends to be possible only with additional consideration of geometric properties like structural compactness and interactions between residues.

As a result of biological complexity and uncertainty in evolutionary deductions, a single domain definition might fail to address the real process of a domain's origin. Additionally, due to the limitations of contemporary automated sequence and structure analysis methods, such a single domain definition may not even be desirable. Instead, we treat domain definitions in 3 categories; two of them (“by sequence” and “by structure”) represent different plausible mechanisms of domain origin based on the same evolutionary considerations. A third category of “composite” domains is based only on geometric properties and not on our evolutionary considerations. We also provide an additional category of “extensions” to list the causative polypeptide segments that differentiate the “by sequence” and “by structure” definitions. Despite this special treatment, the majority of domains do not differ in “by sequence” and “by structure” definitions due to the absence of extensions. Nonetheless, we list them separately in our database for ease of use by automated methods. Thus, our work manages to bring forth a complete and consistent picture of domain structure from an evolutionary perspective for many topologically complex proteins.

An apparently fixed relative arrangement of domains is essential for function in many proteins. However, achieving this fixed positioning may be difficult by a single linker between them. Accordingly, one domain frequently includes a terminal extension that reaches to the neighboring domain, providing stability by additional interactions. This extension can be visualized structurally as part of the interacting domain. From an evolutionary perspective, including the extension as part of the interacting domain presumes a scenario of domain insertion. An alternate scenario can also be envisioned wherein the extension may have evolved from the protruding domain, providing sequence-continuous domain definitions. In most cases the real evolutionary mechanisms that lead to this situation remain unclear: i.e. domain insertion vs. addition of the extension to one of the domains. Consistent resolution of this uncertainty leads to our alternate domain definitions; e.g. “by structure” and “by sequence”. The terms “by sequence” and “by structure” refer only to the attribution of terminal extension to domains, not to the method of domain identification. **First**, in definitions “by structure”, the extensions are assigned to the interacting domain, by structure. This results in a discontinuous sequence for the “ancestral” domain (fig 1b) and implies that one domain was inserted into another. Even if this evolutionary scenario is inaccurate, the structural definition remains meaningful, as the extension frequently occupies a location in the structure that houses the same secondary structural element in a structurally similar single domain protein. Thus, treating the extension as part of the interacting domain may be useful for structure based similarity searches and remote homology inference. **Second**, in definitions “by sequence”, the extension is assigned sequence continuous to the domain it extends from. This definition leads to fewer chain-breaks within domains. Consequently, the “by sequence” definition leads to minimally less compact and globular structural domains than our “by structure” definitions. Keeping sequence continuity and having un-gapped domain sequences makes “by sequence” domain definitions suitable for sequence analysis. Many SCOP [5] domain definitions, outside the multi-domain proteins class, match our “by sequence” definitions, as SCOP tends to maximize sequence continuity of domains. **Third**, we list all of these terminal extensions separately in the database for ease of identification and analysis.

Several extensions and insertions may come together and form a compact region, with a clearly defined hydrophobic core. These regions may often contain closely interacting secondary structural elements and may even share a single β sheet. According to us, these regions do not form a domain, as they are not evolutionarily mobile units, but only a geometric association, arising from extensions to other domains. These compact regions are easily identifiable visually and are often defined as domains by automated domain-definition methods. Therefore, we established a **fourth** category, called composite domains, to accommodate these regions. In addition to being formed from extensions and insertions, some composite domains also include inserted non-globular domains. In all cases, composite domains are sequence discontinuous but form compact spatial bodies that cannot have originated from a single evolutionary event, such as a domain insertion. Thus, these domains are purely geometric and, in our definitions, do not carry evolutionary meaning. We define them simply because they appear in stable conformations, and it seems plausible that similar topologies could be detected as an evolutionary domain in other proteins in the future.

Our alternate domain definitions provide differing perspectives towards domain origin from a few general principles. Since real evidence of domain origin is either unattainable or incomplete, we chose not to limit ourselves to a single optimum domain definition. Further, recent developments in computational resources suggest that merging more accurate sequence and structure searches starting from differing sets of queries will not be limiting in the near future.

### Manual domain definition

We've chosen a set of proteins that is most challenging from the domain definition perspective: the entire SCOP [5] “multi-domain proteins” class. SCOP unifies these proteins in one class as they contain topologically dissimilar domains (e.g. all-alpha, all-beta, alpha/beta and alpha+beta) closely associated with each other, both structurally and functionally. Thus, a “fold” of these proteins is defined as a multi-domain structure, even if it is possible to attribute the individual domains to other SCOP folds. As a result, SCOP does not provide domain boundaries for these proteins, although it frequently mentions domain types and their possible classifications. SCOP domain definitions are a reliable reference; however, considering structures in the multi-domain protein class as single domains is incorrect due to the absence of residue boundaries. Our database gives a reference set of carefully defined domain boundaries for a representative set of these topologically complex proteins. The number of structure representatives in our dataset match closely with the number of entries at the “protein domain” level of the SCOP hierarchy. Thus, we believe our database covers all potential domain arrangements catalogued in the SCOP multi-domain proteins class.

Independent human experts define most domains similarly [24]. This observation implies that biologically meaningful domain definitions require experienced judgments, and contrasts with the pronounced disagreement between results from automated methods. We observed such disagreements during the current work. In our domain definitions, we err towards smaller domains rather than merging several polypeptide segments into larger domains. For novel and unusual domains we emphasize smaller size duplications and small (20–40 residues) geometric formations with defined hydrophobic cores. However, we refrained from splitting well-known domains, such as the Rossmann fold unit, into smaller parts, e.g. into two duplicates forming the doubly-wound fold.

During our manual domain definitions we repeatedly performed certain steps. Although not all of these steps were required for defining every domain, they were observed to be frequent enough to elicit special mention. We discuss them in the approximate order in which they were invoked. 1) Detect structurally similar proteins using structure and sequence methods. Similar but non-identical proteins (homologs) showed differing domain arrangements. Alignments of these homologs indicated domain boundaries for at least some of the domains, making delineation of remaining domains easier. Dissimilar proteins (with a low similarity score) were sometimes observed to contain a similar domain, which we could then align and delineate. Some homologous structures showed additional domains at various stages of growth. To be consistent, we defined these additional domains only if a hydrophobic core was discernable, otherwise the polypeptide segments were treated as loops. 2) Locate possible duplications within a given protein. Structural repeats are likely formed by duplications and thus represented separate evolutionary domains. 3) Attempt to recognize canonical domains like Rossmann fold, ferredoxin-like fold, 4-helical bundle, immunoglobulin, SH3 and OB barrels, etc. This step was limited by our experience. However, frequent referral to relevant literature alleviated some of the problem. The only solution to these limitations is to actually have a reference set of domains, exactly what this current work takes a step towards addressing. Such a reference set could even be used during a structure search in step 1 above. 4) Identify potential domains based on the presence of separate β-sheets. Individual domains most often contained β-sheets in their entirety. In rare cases domains contained more than one sheet, which were however, sequence-intertwined (e.g. beta-sandwich). 5) Identify potential domains based on spatial aggregations of α-helices. Identification of a domain core via step 4 and 5 was usually easy. However position of a domain interface was sometimes unclear. Difference between Intra and inter-domain residue contacts was sometimes difficult to perceive. Side-chain orientation of residues was of some help in this regard. In rare cases we turned to structural similarity detection (step 1 and 3) for help. 6) Treat domains as evolutionary modules by noting sequence continuity and domain insertion events. A pre-requisite for our domain definitions was that all sequence-discontinuous domains could only originate due to other domain insertions. Most automated domain definition methods were observed to fail at this step. 7) Attribute any unassigned peptide segments to the already identified domain cores. Short (<20 residue) segments were attributed to existing domains. Longer segments were either defined as novel domains or attributed to existing domains based on the presence of a perceived hydrophobic core. 8) Exact domain boundaries were refined based on side-chain orientations and interactions as well as superposition of structurally similar domains.

### Potential applications for the domain database

Several applications of our domain database are possible. 1) The database can be used to train and test automated domain definition algorithms as it is the only database that provides domain definitions for the, topologically, most challenging protein chains. 2) The database can be used to study domain interactions, interfaces and topology. Additionally the variation in domain combinations allows the database to be a reference for possible domain architectures. 3) Our manual domain definition criteria, observations and pitfalls may be helpful in the design of an automated domain definition algorithm that considers modular evolutionary units as domains. 4) The compact nature of our structural domains indicates possible folding units or nuclei, and their analysis could enhance understanding of protein folding and structure prediction techniques. 5) Biologists interested in specific proteins catalogued in our database can infer functional and evolutionary units that could be isolated for biochemical studies. 6) Although our composite domains are assembled from several sequence segments that do not suggest mobile evolutionary units, such assembled domains might resemble evolutionary domains in other proteins. As a future work, such findings may yield analogous domain pairs useful for the analysis of convergence in protein evolution.

## Materials and Methods

### Dataset preparation

#### Structure dataset

A dataset of 157 PDB [20] chains representing the SCOP [5] “multi-domain proteins” class (version 1.73) was used for this work. Each structure representative was selected from a single clustered set provided by ASTRAL [25]. ASTRAL provided these clustered sets starting from PDB chains in SCOP with less than 40% sequence identity. Modified residue names in the PDB files (indicated by the MODRES record identifier) were converted to the corresponding residue names of the standard genetic code. Secondary structure information was generated by PALSSE [26] and incorporated into the PDB files.

#### Putative Domain dataset (from CATH [6] and automated methods)

Domain boundaries defined by CATH (version 3.0) were obtained for reference and comparisons. Structural domain assignments by PDP [17] and Domak [16] also assisted quick identification of potential domains. Domak was run with several non-default parameters. The parameters were selected based on manual observation of domains that Domak defined for 100 randomly selected structures from the PDB. Four of these parameters were for increased residue coverage and were set to identical values (MIN_PEAK_BLO_C = MIN_PEAK_SS_ONLY_BLO_C = MIN_PEAK_BLO_DC = MIN_PEAK_SS_ONLY_BLO_DC = 80). Minimum fraction of intra-domain contacts (“Split value”) was increased to obtain more distinct domains (MIN_PEAK_C = MIN_PEAK_DC = MIN_PEAK_MC = 15). Finally, the minimum fraction of secondary structure content required above which secondary structure contacts (as opposed to residue contacts) would be exclusively used was increased (MIN_SS_PER = 0.8).

### Examination of domain characteristics

Visual inspection of the structure coordinates was essential in identifying properties important for domain definition. A number of structural characteristics helped to define the domain boundaries. The most important of these characteristics were secondary structure packing and topology, globularity [3], [7], and hydrophobic cores [27]. Additionally, assessment of structural similarity and evaluation of evolutionary modules helped locate conserved domains and define domain boundaries [10], [12]. Comparison of resulting manual domain definitions with those obtained from available databases, automated methods and published literature helped refine our definitions for difficult cases.

### Manual Domain Definition Procedure

Our method can be broadly split into two steps; an initial step (step 1) of identifying the number and general position of the domains, and (step 2) a later refinement of the domain boundaries. Since refinement of domain boundaries (step 2) also involves assignment of structural extensions to a domain, this step influenced the sequential arrangement and modular representation of our domains. This sequential rearrangement sometimes necessitated changes in domain numbers (step 1) due to our view of domains being capable of modular rearrangement. Thus, we followed an iterative method of domain definition via the above- mentioned two broad steps.

#### Identifying number and general position of domains

The putative domain dataset (described above) was helpful in informing us of potential domains. In most cases structurally distinct compact regions were readily split into domains by automated methods. We considered these putative domains as domain cores and attempted to re-define domains arising from these cores. However, automated methods sometimes showed obvious errors in domain detection. Large and easily recognizable folds like Rossmann, and PIN domain were sometimes over split. For these recognizable folds, our consideration of domain cores was altered from those suggested by the automated methods. In complement, smaller folds in close proximity such as ferredoxin-like, RNaseH, 4-helical bundle, immunoglobulin, SH3 and OB barrels were merged by automated methods and more than one fold was assigned a single domain. For these folds, we relied on visual assessment of secondary structure packing and perceived presence of hydrophobic regions for identification of structurally compact and globular domain cores. Small zinc binding domains were detected by the presence of metal ions and proximal histidine and cysteine residues. Neighboring folds were scrutinized with respect to topology for evidence of duplication. Duplicated domains within a structure were defined by manual observation and sequence and structural alignment.

#### Refinement of domain boundaries

Domain boundaries were refined to assign domains that were modular both by sequence and structure. The possibility that these modules may rearrange during protein evolution was considered, and is expressed in our definitions. Boundary refinement was aided by comparing all similar domains within the same SCOP [5] superfamily in our dataset, which we considered evolutionarily related. Similarity of the potential domains was assessed from structural alignments generated by DaliLite [28], wherein we considered the aligned residues to define conserved regions of a domain. Thus, domain boundaries were set to be consistent between structurally similar and, presumably, evolutionarily related domains. Wherever similar domains were unavailable, domain boundaries were determined by observing polypeptide backbone and residue side-chain geometry. Our assumption that modular domains rearrange during protein evolution played a role in determining domain boundaries. At domain insertion sites, geometry of the polypeptide backbone was studied to assign spatially proximal residues to the ancestral domain. Further, we assumed greater side-chain interaction between intra-domain rather than inter-domain residues.

#### Sequence continuity and alternate domain definitions

Our adherence towards modular domain definitions ensured sequence continuity for individual domains; unless additional inserted domains also were present (except for composite domains described later in this section). Thus for *n* domains inserted into a domain *A*, domain *A* was defined to be composed of *n*+1 polypeptide segments. However, for some pairs of neighboring domains one of the domains was observed to contain terminal structural-extensions that interacted with the neighboring domain. Alternate definitions have been provided wherever these extensions were identified as a secondary structural element (α helix or β sheet). We defined the terminal extension “*by structure*” ensuring structural compactness of the extension to the domain core. This consideration led to a definition where one domain was inserted into the other. Additionally, we defined the terminal extenson “*by sequence*” ensuring sequence continuity of each domain. This alternative led to a definition where the domains appeared terminally fused by sequence, with no discontinuity in either. *Composite* domains form a third category of our domain definitions and are based only on structural compactness. In rare cases, secondary structural extensions and inserts to domains, as well as non-globular domains formed a structurally compact region, wherein a hydrophobic core could be perceived. We define these regions as composite domains although they are sequence discontinuous but do not contain inserted domains.
